# High-Quality, Chromosome-Level Reference Genomes of the Viviparous Caribbean Skinks *Spondylurus nitidus* and *S. culebrae*

**DOI:** 10.1093/gbe/evae079

**Published:** 2024-04-15

**Authors:** Danielle Rivera, James B Henderson, Athena W Lam, Nathan J Hostetter, Jaime A Collazo, Rayna C Bell

**Affiliations:** North Carolina Cooperative Fish and Wildlife Research Unit, Department of Applied Ecology, North Carolina State University, Raleigh, NC, USA; Department of Herpetology, California Academy of Sciences, San Francisco, CA 94118, USA; Center for Comparative Genomics, Institute for Biodiversity Science and Sustainability, California Academy of Sciences, San Francisco, CA 94118, USA; Center for Comparative Genomics, Institute for Biodiversity Science and Sustainability, California Academy of Sciences, San Francisco, CA 94118, USA; U.S. Geological Survey, North Carolina Cooperative Fish and Wildlife Research Unit, Department of Applied Ecology, North Carolina State University, Raleigh, NC, USA; U.S. Geological Survey, North Carolina Cooperative Fish and Wildlife Research Unit, Department of Applied Ecology, North Carolina State University, Raleigh, NC, USA; Department of Herpetology, California Academy of Sciences, San Francisco, CA 94118, USA

**Keywords:** viviparous, Caribbean, genome assembly, skink, *Spondylurus*

## Abstract

New World mabuyine skinks are a diverse radiation of morphologically cryptic lizards with unique reproductive biologies. Recent studies examining population-level data (morphological, ecological, and genomic) have uncovered novel biodiversity and phenotypes, including the description of dozens of new species and insights into the evolution of their highly complex placental structures. Beyond the potential for this diverse group to serve as a model for the evolution of viviparity in lizards, much of the taxonomic diversity is concentrated in regions experiencing increasing environmental instability from climate and anthropogenic change. Consequently, a better understanding of genome structure and diversity will be an important tool in the adaptive management and conservation of this group. Skinks endemic to Caribbean islands are particularly vulnerable to global change with several species already considered likely extinct and several remaining species either endangered or threatened. Combining PacBio long-read sequencing, Hi-C, and RNAseq data, here we present the first genomic resources for this group by describing new chromosome-level reference genomes for the Puerto Rican Skink *Spondylurus nitidus* and the Culebra Skink *S. culebrae*. Results indicate two high quality genomes, both ∼1.4 Gb, assembled nearly telomere to telomere with complete mitochondrion assembly and annotation.

**Significance**

Skinks in the subfamily Mabuyinae are unique, diverse, and understudied organisms ranging across the Caribbean and Central and South America. Species within the genus *Spondylurus* are of current conservation concern and distributed across threatened habitats on Antillean islands. We provide high quality, chromosome-level reference genomes for two *Spondylurus* species, which will serve as an invaluable resource for future analyses of biodiversity and conservation in this and related species groups.

## Introduction

New World mabuyine skinks are a radiation of 227 currently described species and 26 genera distributed across the Caribbean and Central and South America (Uetz et al. 2021). Their conserved morphologies resulted in few species being recognized historically but more recent studies combining population-level morphological, ecological, and genomic data have revealed novel biodiversity resulting in the description of a number of independent lineages as new taxa (Hedges and Conn 2012; Pinto-Sánchez et al. 2015). In parallel, more focused studies have provided key insights into the evolution of their highly complex placental structures (Cornelis et al. 2017). Consequently, this diverse group has the potential to serve as a model for understanding the ecological context and evolutionary mechanisms by which viviparity has evolved in tropical lizards. There are currently no genomic resources available within the mabuyine radiation, few genomic resources are available for the higher order, family level group Scincidae, which consists of over 1,700 species (Uetz et al. 2021), and no genomic resources are available for any viviparous skink species (https://www.ncbi.nlm.nih.gov/datasets/genome/?taxon=66056). Furthermore, much of the diversity in this group is concentrated in regions experiencing increasing environmental instability from climate and anthropogenic change (Myers et al. 2000). Consequently, a better understanding of genome structure and diversity in this group will also be an important tool to inform potential adaptive management measures such as genetic rescue and reintroductions.

Tropical reptiles are thought to be particularly vulnerable to potential extinctions from climate change (Huey et al. 2012; Sinervo et al. 2010). Mabuyine skinks in the genus *Spondylurus*, described in 2012 and distributed across the Caribbean, are considered rare and of increasing conservation concern (https://www.iucnredlist.org/search/list?query=spondylurus&searchType=species; IUCN 2022). Of the 17 described species, one is considered Near Threatened, three are considered Endangered, and 13 are considered Critically Endangered (Hedges and Conn 2012; IUCN 2022). At present, there are few ecological or genomic resources available to aid in the conservation and recovery of these species. Here we present the first genomic resources for this group by describing new annotated, chromosome-level reference genomes for the Puerto Rican Skink *Spondylurus nitidus* and the Culebra Skink *S. culebrae*.

## Results and Discussion

### Genome Assembly and Annotation

**Fig. 1.**
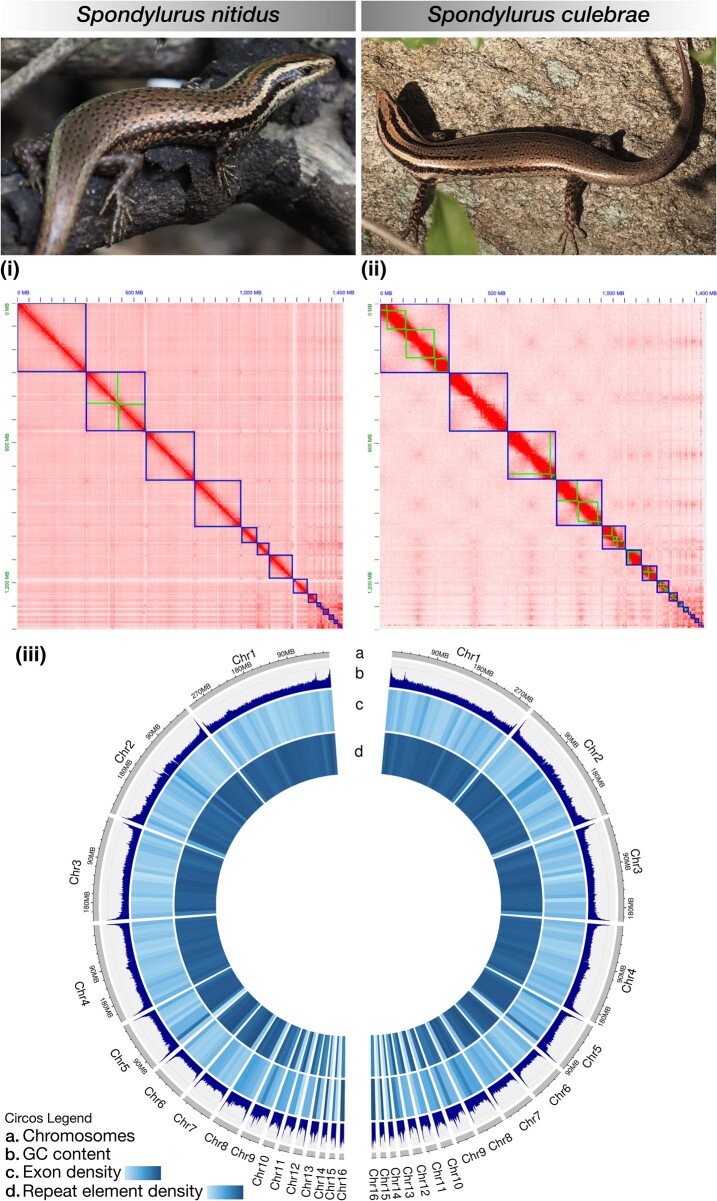
(i) Hi-C Juicebox contact heatmap for *Spondylurus nitidus* and (ii) for *S. culebrae* (small squares: scaffold annotations; large squares: chromosomes); (iii) Circos plot indicating a) chromosome lengths, b) GC content in 500 kb windows, c) gene density (segments per 10 Mb), d) repeat element density (segments per 10 Mb) for *S. nitidus* (left) and *S. culebrae* (right). Photographs by J.P. Zegarra (*S. nitidus*) and N. Arocho-Hernandez (*S. culebrae*).

For *S. nitidus* (voucher number UPRM-R1239), the three HiFi cells generated 7,941,060 raw reads, having N50 read lengths 13,135 bp, 13,128 bp, and 12,786 bp. After cleanup, 7,939,771 reads were used for the contig assembly (Table 1, supplementary table S1, Supplementary Material online). Telomeres were found on at least one end of 18 contigs and 13 contigs had a top and bottom telomere in the HiFi read only assembly. The hifiasm assembly resulted in 1.398 Gb across 28 contigs, with a scaffold N50 = 199.7 Mb (Table 1). Using the interactive JuiceBox Assembly Tools (JBAT) modification with the YaHS scaffolded assembly and the Hi-C reads yielded an assembly with 16 chromosomal records, 15 telomere to telomere and the 16th with a single telomere (Fig. 1). The final assembly was very high quality, as evidenced by 98.1% completeness (compleasm; Table 1). In agreement with previous karyotypic analysis of closely-related species (2N = 32), the categorization of chromosome lengths into four groups broadly agrees with the 16 chromosome sizes seen in the *S. nitidus* assembly (Fig. 1; Adegoke and Ejere 1991). Repeatmasker found 44.99% of the genome to be repeat elements (Fig. 1, supplementary table S5, Supplementary Material online). BRAKER3 identified 18,534 annotated genes out of 19,207 total genes (96.5% completedness), with 201,278 Exons (mean length = 170.94), 179,398 Introns (mean length = 2,218.28), and 21,880 mRNA (mean length = 20,001.64) (Table 1, supplementary tables S3 and S4, Supplementary Material online).

**Table 1:** *Spondylurus nitidus* and *S. culebrae* sequencing, assembly, and gene annotation read statistics

			*S. nitidus*	*S. culebrae*
PacBio HiFi	Total	Reads	7,941,060	5,367,405
		Bases	103,521,825,924	65,628,464,440
		Post-cleanup reads	7,939,771	5,364,894
		Post-cleanup bases	103,509,184,716	65,626,492,310
Hi-C		Read pairs	78,871,462	61,820,420
Assembly		No. of scaffolds	20	16
		No. of scaffolds > 1M	16	16
		Contig N50 (Mb)	199.70	85.72
		Scaffold N50 (Mb)	208.67	207.72
		GC %	43.54	43.53
		Repeat elements (%)	44.99	44.96
RNAseq	Gene annotation (% of genome)	Total genes	19,207	19,445
		Annotated genes	18,534 (25.43%)	18,494 (38.35%)
		Gene annotation completeness	96.5%	95.11%
		Exons	201,278 (2.46%)	267,703 (3.26%)
		Introns	179,398 (28.47%)	243,311 (52.33%)
		mRNA	21,880 (31.31%)	24,392 (56.94%)
BUSCO/compleasm (Sauropsida lineage)		Total groups searched	7,480/7,480	7,480/7,480
		Complete single-copy	7,009 (93.7%)/7,317 (97.82%)	7,006 (93.7%)/7,314 (97.78%)
		Complete duplicated	78 (1.0%)/21 (0.28%)	87 (1.2%)/24 (0.32%)
		Fragmented	83 (1.1%)/29 (0.39%)	82 (1.1%)/27 (0.36%)
		Missing	310 (4.2%)/113 (1.51%)	305 (4.0%)/115 (1.54%)

For *S. culebrae* (voucher number UPRM-R1240), the two HiFi cells generated 5,367,405 raw reads, having N50 read lengths 12,661 bp and 12,448 bp. After cleanup, 5,364,894 reads were used for the contig assembly (Table 1). This was ∼two-thirds of the base coverage in the *S. nitidus* assembly with slightly lower N50 read length. The assembly had a very balanced use of reads with 52% of the reads from one cell's output and 48% from the other. The resultant assembly of 76 contigs was used in YaHS with the Hi-C data and resulted in an assembly of 41 records, the 16 largest of which were identified as chromosomes (Table 1, Fig. 1). Six of the purge_dups haplotig designated records had BUSCOs found but all of them were duplicates found in the chromosome sequences. As a result we removed all but the 16 records designated as chromosomes from the final assembly. All but one chromosomal record have at least one telomere and 12 have both telomeres, with a genome size of 1.391 Gb, N50 = 207.7 Mb, and 98.1% completeness (compleasm; Table 1, supplementary S1, Supplementary Material online). Repeatmasker found 44.96% of the genome to be repeat elements (Fig. 1, supplementary table S5, Supplementary Material online). The number of bases, BUSCO/compleasm score, repeat percentage of the genome assembly, and distribution of repeat types is very similar to the metrics from the *S. nitidus* assembly (supplementary table S5, Supplementary Material online). BRAKER3 identified 18,494 annotated genes out of 19,445 total genes (95.11% completedness), with a total of 267,703 Exons (mean length = 169.48), 243,311 Introns (mean length = 2,992.82), and 24,392 mRNA (mean length = 32,483.50) (Table 1, supplementary tables S3 and S4, Supplementary Material online).

### Mitochondrion Assembly and Annotation

The *S. nitidus* consensus mitochondrion sequence derived from 175 mitochondrial HiFi reads was 16,990 bp with 22 tRNAs, 13 protein coding genes, and two rRNAs, as typical in vertebrates. The control region of 1,610 bp had significant repeat regions of 44 and 60 bp repeated 3.9 and 4.5 times, respectively, and a 4 bp repeat occurring 16 times. The heteroplasmic analysis of 125 records containing the control region found an additional layout supported by 36 mitochondrial HiFi reads differing from the consensus by the number of 44 bp repeated motifs at 2.9 copies. The *S. culebrae* consensus mitochondrion sequence derived from 144 mitochondrial HiFi reads was 16,821 bp with 22 tRNAs, 13 protein coding genes, and two rRNAs. The control region of 1,445 bp had the same repeat regions as *S. nitidus*, repeated 1.9 and 3.6 times, respectively, and a 4 bp repeat occurring 15.8 times. The consensus motifs are nearly identical to those found in the *S. nitidus* mitochondrion.

## Materials and Methods

### Sampling

Skinks were captured from field sites using lassos and euthanized following approved IACUC protocols. *Spondylurus nitidus* was collected from Guajataca State Park in northwestern Puerto Rico, and *S. culebrae* was collected from Monte Resaca within the Culebra National Wildlife Refuge on the island of Culebra, Puerto Rico. Liver, muscle, blood, and other organs were collected from both individuals and stored in RNALater (Thermo Fisher Scientific), PGShield (Phase Genomics), or were fresh-frozen. Voucher specimens were fixed in formalin and deposited in the Herpetology Collection at the University of Puerto Rico at Mayagüez.

### HiFi, Hi-C, and RNAseq Library Prep

High molecular weight (HMW) DNA was extracted from multiple tissues from both species using phenol-chloroform (PCI) for long-read sequencing following the PacBio recommended protocol (https://www.pacb.com/wp-content/uploads/2015/09/SharedProtocol-Extracting-DNA-usinig-Phenol-Chloroform.pdf). An additional round of SPRI bead cleaning was done to eliminate impurities to meet the DNA requirement for PacBio sequencing. DNA concentration was determined with the Qubit dsDNA HS Assay Kit (Invitrogen Corp.), and high molecular weight content was confirmed by running a Femto Pulse (Agilent). DNA was sent to Maryland Genomics for HiFi library preparation and sequencing with PacBio Sequel II SMRT Cells (*S. culebrae*: two cells; *S. nitidus*: three cells).

For *S. nitidus*, a Hi-C library was prepared from muscle tissue, and for *S. culebrae*, a library was prepared from blood. Tissues were homogenized using sterile razor blades prior to fixation. In situ Hi-C libraries were prepared as described in Rao et al. (2014) with modifications. Briefly, after Streptavidin Pull-down, the biotinylated Hi-C products underwent end repair, ligation, and enrichment using the NEBNext Ultra II DNA Library Preparation Kits (New England Biolabs Inc). Then, titration of the number of PCR cycles was performed as described in Belton et al. (2012). Libraries were then sent to Novogene for paired-end (PE)150 sequencing on an Illumina NovaSeq.

Whole muscle tissue from *S. nitidus* stored in RNALater was sent to Novogene for RNAseq library prep. RNA from *S. culebrae* was extracted from liver and lung tissue stored in RNALater and PGShield, respectively, using the NEB Monarch RNA Cleanup Kit (New England Biolabs). These RNA were prepped for RNAseq using the Illumina Total RNA Prep with Ribo-Zero Plus Kit (Illumina, CA, USA). All RNA sequencing was performed PE150 on an Illumina NovaSeq at Novogene.

### Reference Genome Assembly

We filtered reads from the HiFi (*S. nitidus*: three SMRT cells; *S. culebrae*: two SMRT cells) fastq files using cutadapt v4.4 (Martin 2011) removing any reads less than 1,000 bp in length or that contained a PacBio SMRTbell adapter in any position. To assess genome sizes, we ran Jellyfish v2.3.0 (Marçais and Kingsford 2011) using a kmer size of 21, and generated histograms of kmer frequencies for each species. We then used the GenomeScope2 website to provide estimates of genome properties including total size, repeat content, and heterozygosity (Ranallo-Benavidez et al. 2020). To assemble genomes, we used HiFiasm with the HiFi cleaned fastq read sets to create a contig level assembly (Cheng et al. 2021). The HiFiasm gfa output was converted to fasta files using gfatools (https://github.com/lh3/gfatools; Li et al. 2020), and circular records were extracted into separate files. Basic statistics were calculated for the contig level assemblies, and BUSCO v5.4.7 runs were initiated using the sauropsida lineage (Simão et al. 2015).

For scaffolding, the Hi-C reads from muscle tissue (*S. nitidus*) and blood (*S. culebrae*) were cleaned and prepared in two steps: first, fastp v0.23.2 (Chen et al. 2018) was used to remove Illumina adapters and any reads less than 100 bp; then the dedup argument was applied in addition to the default settings; second, we used Arima's pipeline (github.com/ArimaGenomics/mapping_pipeline) for additional cleanup and mapped the reads to the contig assemblies with BWA (Li 2013). The resultant bam files were used as input with the contig assembly in the YaHS scaffolding program (Zhou et al. 2023). YaHS was run twice, with and without the default use of the Hi-C read coverage to perform contig splitting. The versions without correction were chosen for further analysis of both species’ genomes. We then used Juicebox Assembly Tools (JBAT) v1.11.08 (Durand et al. 2016) for manual refinement, and interactively updated the location and orientation of contigs and their delineation within chromosomes. These assemblies were also queried against the nt database using blastn to identify any contaminants for removal.

To assess the level of genome completeness, we ran both compleasm v0.2.2 (Huang and Li 2023), a reimplementation of BUSCO using miniprot (Li 2023) and BUSCO v5.4.7 with its default MetaEuk (Levy Karin et al. 2020) mode using the sauropsida lineage (best lineage determined by BUSCO; supplementary table S2, Supplementary Material online).

### Reference Genome Annotation

Prior to gene annotation, de novo repeats were identified using RepeatModeler v2.0.1 (Flynn et al. 2020). The repeat models found from the assemblies and the vertebrate repeat models from Repbase RepeatMasker libraries were combined and used in Repeatmasker v4.0.9 (Smit et al. (2013) to create a soft-masked repeat version of the assembly file used for the gene model structural annotation and to construct a table of repeat types and lengths. We then used BRAKER3 (Gabriel et al. 2023), which runs using the RNA sequences mapped with HiSat2 to the genome assembly, and then GeneMark-EP+ and AUGUSTUS combined with the vertebrate protein database from OrthoDB v10 (Stanke et al. 2008, 2006; Hoff et al. 2019; Brůna et al. 2021) to annotate the genomes. The resulting gff3 annotation files, coding sequence DNA files, and protein sequence Amino Acid files were then used as input for further refinement and functional annotation.

We began functional annotation of both genomes by looking for protein domains in the amino acid sequences using BRAKER3 v3.0.3 by running InterProScan-5.61-93.0 (Jones et al. 2014). The sequences were blasted against GenBank using blastn (nt database), blastp with SwissProt, diamond blastp (Buchfink et al. 2021) with the TrEMBL database, and diamond blastp with OrthoDB11 vertebrate. Protein domain IDs and Gene Ontology terms from the InterProScan output were added to the gff3 file for each gene model as was the functional annotation description from the lowest eValue for each gene from the blast searches. Sequences were then aligned using STAR v2.7.10b (Dobin et al. 2013) and HISAT2 v2.2.0 (Kim et al. 2019).

Output files (gff3, scaflens, fasta) from scaffolding, BRAKER, and RepeatModeler were converted and used as input files for Circos plots using custom python scripts (https://github.com/daniellerivera/Spondylurus) in shinyCircos v2.0 (Wang et al. 2023).

### Mitochondrial Genome Assembly

The HiFiMiTie pipeline (github.com/calacademy-research/HiFiMiTie) using the HiFi read input and Squamata taxid 8509 was used to pull mitochondrial records from the HiFiasm error-corrected reads to construct a consensus mitochondrion, its annotation, and analysis of control region heteroplasmy for both reference genome assemblies. Taxid was used to define the canonical RNA and was used to choose the appropriate genetic code for annotation and to select mitogenomes from the NCBI mitochondrial database to blast against. To discover additional mitochondrial reads where no current genome has similar control region structure or repeat motifs, mitochondrial records found from the initial blast search of the Squamata mitogenomes were used to query the HiFi reads again. MITOS2 (Bernt et al. 2013; Donath et al. 2019) was then used to confirm annotations.

## Supplementary Material

evae079_Supplementary_Data

## Data Availability

Data presented in this article are available from NCBI (Project PRNJA1039818; https://www.ncbi.nlm.nih.gov/bioproject/PRJNA1039818). Bioinformatics code and additional genome files are available on GitHub (https://github.com/calacademy-research/, https://github.com/daniellerivera/Spondylurus). Data availability questions can be directed to the corresponding author (DR) via email drivera2288@gmail.com.
