# Supplementary material for: High-Quality, Chromosome-Level Reference Genomes of the Viviparous Caribbean Skinks Spondylurus nitidus and S. culebrae
Source: Genome Biol Evol. 2024 Apr 15;16(5):evae079. doi: 10.1093/gbe/evae079 (PMC11065358; doi:10.1093/gbe/evae079)
Supplement: evae079_Supplementary_Data [file evae079_supplementary_data.docx]

**High-quality, chromosome-level reference genomes of the viviparous Caribbean skinks *Spondylurus nitidus* and *S. culebrae***

Danielle Rivera^1,4,*^, James B. Henderson^2^, Athena W. Lam^2^, Nathan J. Hostetter^3^, Jaime A. Collazo^3^, Rayna C. Bell^4^

1. North Carolina Cooperative Fish and Wildlife Research Unit, Department of Applied Ecology, North Carolina State University, Raleigh, North Carolina, USA
2. Center for Comparative Genomics, Institute for Biodiversity Science and Sustainability, California Academy of Sciences, San Francisco, California 94118, USA
3. U.S. Geological Survey, North Carolina Cooperative Fish and Wildlife Research Unit, Department of Applied Ecology, North Carolina State University, Raleigh, North Carolina, USA
4. California Academy of Sciences, Department of Herpetology, San Francisco, California 94118, USA

*corresponding author: e-mail: drivera2288@gmail.com

# Supplementary Material

Any use of trade, firm, or product names is for descriptive purposes only and does not imply endorsement by the U.S. Government.

## Genome Assembly and Annotation Results

For *Spondylurus nitidus*, the GenomeScope 21mer modeling and the hifiasm assembly both estimated a 75X homozygous and 37X heterozygous coverage with just under 0.6% heterozygosity, indicating very high quality results even before scaffolding with Hi-C data. For *S. culebrae*, the GenomeScope 21mer analysis and the HiFiasm assembly both showed homozygous kmer coverage peak of 49X and heterozygous peak of 25X.

**Table S1. Expanded *Spondylurus nitidus* and *S. culebrae* sequencing, assembly, and gene annotation read metrics.**

|  | | | ***S. nitidus*** | ***S. culebrae*** |
| --- | --- | --- | --- | --- |
| PacBio HiFi | Cell 1 | reads | 3,002,435 | 2,714,930 |
|  |  | bases | 39,426,172,667 | 33,432,378,921 |
|  |  | N50 read length | 13,135 | 12,661 |
|  | Cell 2 | reads | 2,645,899 | 2,652,475 |
|  |  | bases | 34,700,496,183 | 32,196,085,519 |
|  |  | N50 read length | 13,128 | 12,448 |
|  | Cell 3 | reads | 2,292,726 | - |
|  |  | bases | 29,395,157,074 | - |
|  |  | N50 read length | 12,786 | - |
|  | Total | reads | 7,941,060 | 5,367,405 |
|  |  | bases | 103,521,825,924 | 65,628,464,440 |
|  |  | post-cleanup reads | 7,939,771 | 5,364,894 |
|  |  | post-cleanup bases | 103,509,184,716 | 65,626,492,310 |
| Hi-C | | read pairs | 78,871,462 | 61,820,420 |
| Assembly | | No. of scaffolds | 20 | 16 |
|  |  | No. of scaffolds > 1M | 16 | 16 |
|  |  | Contig N50 (Mb) | 199.70 | 85.72 |
|  |  | Scaffold N50 (Mb) | 208.67 | 207.72 |
|  |  | GC % | 43.54 | 43.53 |
|  |  | Repeat Elements (%) | 44.99 | 44.96 |
| RNA-seq | Gene Annotation  (% of genome) | Total genes | 19,207 | 19,445 |
|  |  | annotated genes | 18,534 (25.43%) | 18,494 (38.35%) |
|  |  | exons | 201,278 (2.46%) | 267,703 (3.26%) |
|  |  | introns | 179,398 (28.47%) | 243,311 (52.33%) |
|  |  | mRNA | 21,880 (31.31%) | 24,392 (56.94%) |
| BUSCO / compleasm  (Sauropsida lineage) | | Complete | 7,087 (94.7%) / 7,338 (98.10%) | 7,093 (94.9%) / 7,338 (98.10%) |
|  |  | Complete single-copy | 7,009 (93.7%) / 7,317 (97.82%) | 7,006 (93.7%) / 7,314 (97.78%) |
|  |  | Complete duplicated | 78 (1.0%) / 21 (0.28%) | 87 (1.2%) / 24 (0.32%) |
|  |  | Fragmented | 83 (1.1%) / 29 (0.39%) | 82 (1.1%) / 27 (0.36%) |
|  |  | Missing | 310 (4.2%) / 113 (1.51%) | 305 (4.0%) / 115 (1.54%) |
|  |  | Total groups searched | 7,480 / 7,480 | 7,480 / 7,480 |

**Table S2. Busco lineage results**

# looking for full taxonomy of "Scinciformata; Scincidae;"

1273157 | Acontinae | cellular organisms; Eukaryota; Opisthokonta; Metazoa; Eumetazoa; Bilateria; Deuterostomia; Chordata; Craniata; Vertebrata; Gnathostomata; Teleostomi; Euteleostomi; Sarcopterygii; Dipnotetrapodomorpha; Tetrapoda; Amniota; Sauropsida; Sauria; Lepidosauria; Squamata; Bifurcata; Unidentata; Scinciformata; Scincidae; |

# matching busco lineages

eukaryota_odb10 [255]

- metazoa_odb10 [954]

- vertebrata_odb10 [3354]

- tetrapoda_odb10 [5310]

- sauropsida_odb10 [7480]

# best busco lineage

sauropsida

**Table S3. Reference Genome Annotation for *Spondylurus nitidus* and *S. culebrae*.**

|  | *S. nitidus* | *S. culebrae* |
| --- | --- | --- |
| Genes | 19,207 | 19,445 |
| Percentage of Genome | 25.43% | 38.35% |
| Total Gene Length | 355,421,434 | 533,620,992 |
| Assembly Length | 1,397,740,396 | 1,391,542,637 |
| mRNA | 21,880 | 24,392 |
| Mean Length | 20,001.64 | 32,483.5 |
| Longest | 547,632 | 987,993 |
| Shortest | 123 | 123 |
| Total mRNA Length | 437,635,880 | 792,337,631 |
| Exons | 201,278 | 267,703 |
| Mean per mRNA | 9.2 | 10.98 |
| Mean Length | 170.94 | 169.48 |
| Mean Length per mRNA | 1,572.54 | 1,860.06 |
| Longest Exon | 14,867 | 35,577 |
| Shortest Exon | 3 | 1 |
| Total Exon Length | 34,407,180 | 45,370,572 |
| Single Exon mRNA | 2,787 | 2,236 |
| Introns | 179,398 | 243,311 |
| Mean per mRNA | 8.2 | 9.98 |
| Mean Length | 2,218.28 | 2,992.82 |
| Mean Length per mRNA | 18,188.1 | 29,853.51 |
| Longest Intron | 331,887 | 329,817 |
| Shortest Intron | 62 | 20 |
| Total Intron Length | 397,955,671 | 728,186,762 |
| mRNA % of genome | 31.31% | 56.94% |
| exons % of genome | 2.46% | 3.26% |
| introns % of genome | 28.47% | 52.33% |
| Assembly Length | 1,397,740,396 | 1,391,542,637 |
| Genes with gene names | 18,534 | 18,494 |
| annotation completeness | 96.50% | 95.11% |
| mRNA with gene names | 21,190 | 23,418 |
| annotation completeness | 96.85% | 96.01% |
| mRNA with gene descriptions | 21,219 | 23,444 |
| annotation completeness | 96.98% | 96.11% |

**Table S4. STAR (*Spondylurus nitidus*) and HiSAT2 (*S. culebrae*) RNAseq mapping results**

| ***S. nitidus***: 1 tissue type: *Whole Muscle. RNAseq mapping program STAR* | | |
| --- | --- | --- |
| Mapping speed, Million of reads per hour | \| 308.62 | |
| Number of input reads | \| 51778829 | |
| Average input read length | \| 298 | |
| UNIQUE READS: | | |
| Uniquely mapped reads number | \| 42260574 | |
| Uniquely mapped reads % | \| 81.62% | |
| Average mapped length | \| 296.04 | |
| Number of splices: Total | \| 38560334 | |
| Number of splices: Annotated (sjdb) | \| 0 | |
| Number of splices: GT/AG | \| 38257128 | |
| Number of splices: GC/AG | \| 263155 | |
| Number of splices: AT/AC | \| 9915 | |
| Number of splices: Non-canonical | \| 30136 | |
| Mismatch rate per base, % | \| 0.32% | |
| Deletion rate per base | \| 0.02% | |
| Deletion average length | \| 2.50 | |
| Insertion rate per base | \| 0.01% | |
| Insertion average length | \| 1.95 | |
| MULTI-MAPPING READS: | | |
| Number of reads mapped to multiple loci | \| 445266 | |
| % of reads mapped to multiple loci | \| 0.86% | |
| Number of reads mapped to too many loci | \| 2551178 | |
| % of reads mapped to too many loci | \| 4.93% | |
| UNMAPPED READS: | | |
| Number of reads unmapped: too many mismatches | \| 0 | |
| % of reads unmapped: too many mismatches | \| 0.00% | |
| Number of reads unmapped: too short | \| 6388011 | |
| % of reads unmapped: too short | \| 12.34% | |
| Number of reads unmapped: other | \| 133800 | |
| % of reads unmapped: other | \| 0.26% | |
| CHIMERIC READS: | | |
| Number of chimeric reads | \| 0 | |
| % of chimeric reads | \| 0.00% | |
| **82.48% Uniquely and multiple loci map total** | | |
|  | | |
| ***S. culebrae***: 2 tissue types: Lung and Liver. RNAseq mapping program HISAT2 | | |
| *hisat2.SponCul_lung results*: | |  |
| 65590425 reads; | | of these: |
| 65590425 (100.00%) were paired; | | of these: |
| 5656349 (8.62%) | | aligned concordantly 0 times |
| 49039561 (74.77%) | | aligned concordantly exactly 1 time |
| 10894515 (16.61%) | | aligned concordantly >1 times |
|  | |  |
| 5656349 pairs aligned concordantly 0 times; | | of these: |
| 58840 (1.04%) | | aligned discordantly 1 time |
| 5597509 pairs aligned 0 times concordantly or discordantly; | | of these: |
| 11195018 mates make up the pairs; | | of these: |
| 7873544 (70.33%) | | aligned 0 times |
| 2204928 (19.70%) | | aligned exactly 1 time |
| 1116546 (9.97%) | | aligned >1 times |
| 94.00% overall alignment rate | | |
| 74.77% + 16.61% = **91.3%** | | |
| *hisat2.SponCul_liver results*: | |  |
| 61605504 reads; | | of these: |
| 61605504 (100.00%) were paired; | | of these: |
| 5913082 (9.60%) | | aligned concordantly 0 times |
| 41767916 (67.80%) | | aligned concordantly exactly 1 time |
| 13924506 (22.60%) | | aligned concordantly >1 times |
|  | |  |
| 5913082 pairs aligned concordantly 0 times; | | of these: |
| 50608 (0.86%) | | aligned discordantly 1 time |
|  | |  |
| 5862474 pairs aligned 0 times concordantly or discordantly; | | of these: |
| 11724948 mates make up the pairs; | | of these: |
| 8370745 (71.39%) | | aligned concordantly 0 times |
| 1715339 (14.63%) | | aligned concordantly exactly 1 time |
| 1638864 (13.98%) | | aligned concordantly >1 times |
| 93.21% overall alignment rate | | |
| 67.80% + 22.60% = **90.4%** | | |

**Table S5. Repeats Tables**

| SponNit_1.0.fasta | | | | |
| --- | --- | --- | --- | --- |
| sequences: | 26 |  |  | |
| total length: | 1,397,844,510 bp (1,397,844,110 bp excl N/X-runs) | | | |
| GC level: | 43.54% |  |  | |
| bases masked: | 628,834,606 bp (44.99%) | |  | |
|  | number of elements* | length occupied | percentage of sequence | |
| Retroelements | 1,525,233 | 260,245,583 bp | 18.62% | |
| SINEs: | 274,660 | 30,484,795 bp | 2.18% | |
| Penelope | 28,012 | 3,193,702 bp | 0.23% | |
| LINEs: | 1,002,412 | 166,423,834 bp | 11.91% | |
| CRE/SLACS | 0 | 0 bp | 0.00% | |
| L2/CR1/Rex | 629,261 | 94,377,701 bp | 6.75% | |
| R1/LOA/Jockey | 2,835 | 226,564 bp | 0.02% | |
| R2/R4/NeSL | 14,831 | 12,962,778 bp | 0.93% | |
| RTE/Bov-B | 291,456 | 48,595,727 bp | 3.48% | |
| L1/CIN4 | 29,023 | 6,149,531 bp | 0.44% | |
| LTR elements: | 24,8161 | 63,336,954 bp | 4.53% | |
| BEL/Pao | 2,604 | 119,683 bp | 0.01% | |
| Ty1/Copia | 94,460 | 29,925,103 bp | 2.14% | |
| Gypsy/DIRS1 | 44,630 | 8,573,113 bp | 0.61% | |
| Retroviral | 53,761 | 12,614,557 bp | 0.90% | |
| DNA transposons | 1,237,305 | 159,968,982 bp | 11.44% | |
| hobo-Activator | 94,4517 | 128,095,935 bp | 9.16% | |
| Tc1-IS630-Pogo | 136,748 | 20,757,534 bp | 1.48% | |
| En-Spm | 0 | 0 bp | 0.00% | |
| MuDR-IS905 | 0 | 0 bp | 0.00% | |
| PiggyBac | 2,915 | 264,263 bp | 0.02% | |
| Tourist/Harbinger | 20,287 | 1,356,722 bp | 0.10% | |
| Other (Mirage, P-element, Transib) | 161 | 6348 bp | 0.00% | |
| Rolling-circles | 15,250 | 1,462,734 bp | 0.10% | |
| Unclassified: | 1,336,931 | 201,445,183 bp | 14.41% | |
| Total interspersed repeats: | | 621,659,748 bp | | 44.47% |
| Small RNA: | 21,037 | 4,611,320 bp | 0.33% | |
| Satellites: | 16,064 | 1,296,754 bp | 0.09% | |
| Simple repeats: | 2,644 | 200,123 bp | 0.01% | |
| Low complexity: | 0 | 0 bp | 0.00% | |
| * most repeats fragmented by insertions or deletions have been counted as one element | | | | |

| **SponCul_1.0.fasta** | | | |
| --- | --- | --- | --- |
| sequences: | 16 |  |  |
| total length: | 1,391,542,637 bp (1,391,539,137 bp excl N/X-runs) | | |
| GC level: | 43.53% |  |  |
| bases masked: | 625,611,324 bp (44.96%) | | |
|  | number of elements* | length occupied | percentage of sequence |
| Retroelements | 1,432,157 | 251,952,847 bp | 18.11% |
| SINEs: | 267,224 | 30,221,748 bp | 2.17% |
| Penelope | 0 | 0 bp | 0.00% |
| LINEs: | 963,555 | 163,669,612 bp | 11.76% |
| CRE/SLACS | 0 | 0 bp | 0.00% |
| L2/CR1/Rex | 618,454 | 93,880,861 bp | 6.75% |
| R1/LOA/Jockey | 2,794 | 221,097 bp | 0.02% |
| R2/R4/NeSL | 14,477 | 12,494,685 bp | 0.90% |
| RTE/Bov-B | 276,196 | 46,844,717 bp | 3.37% |
| L1/CIN4 | 20,083 | 5,762,999 bp | 0.41% |
| LTR elements: | 201,378 | 58,061,487 bp | 4.17% |
| BEL/Pao | 1,601 | 84,316 bp | 0.01% |
| Ty1/Copia | 93,425 | 28,260,260 bp | 2.03% |
| Gypsy/DIRS1 | 34,004 | 8,162,502 bp | 0.59% |
| Retroviral | 22,097 | 9,612,900 bp | 0.69% |
| DNA transposons | 1,131,779 | 155,218,670 bp | 11.15% |
| hobo-Activator | 908,505 | 126,551,970 bp | 9.09% |
| Tc1-IS630-Pogo | 132,282 | 20,588,843 bp | 1.48% |
| En-Spm | 0 | 0 bp | 0.00% |
| MuDR-IS905 | 25,037 | 16,73,533 bp | 0.12% |
| PiggyBac | 2,412 | 245,396 bp | 0.02% |
| Tourist/Harbinger | 12,153 | 1,050,567 bp | 0.08% |
| Other (Mirage, P-element, Transib) | 85 | 3,879 bp | 0.00% |
| Rolling-circles | 9,973 | 1,311,861 bp | 0.09% |
| Unclassified: | 1,328,482 | 200,578,566 bp | 14.41% |
| Total interspersed repeats: | | 607,750,083 bp | 43.67% |
| Small RNA: | 16,993 | 4,197,116 bp | 0.30% |
| Satellites: | 7,842 | 859,388 bp | 0.06% |
| Simple repeats: | 241,791 | 10,875,967 bp | 0.78% |
| Low complexity: | 19,004 | 860,795 bp | 0.06% |
| * most repeats fragmented by insertions or deletions have been counted as one element | | | |
| RepeatMasker version 4.1.5, sensitive mode | | | |
| run with rmblastn version 2.14.0+ | | | |

## Materials and Methods (additional detail)

***PacBio HiFi Sequencing:***

Tissues were stored in PGshield. Half of the resulting tissues were used for Phenol Chloroform (PCI) based high molecular weight (HMW) DNA extraction for PacBio sequencing (the other half of the material was used as starting material for Hi-C library preparation, see below).

Tissues were homogenized on ice using a sterile razor blade. ATL buffer (140 μl) and Proteinase K (60 μl) were then added to the homogenized material and incubated at 65°C for 1 hr. The 200 μl of resulting lysate was used as starting material for the PCI extraction following a PacBio recommended protocol (PacBio, 2012). An additional round of SPRI bead cleaning was done to eliminate impurities to meet the DNA requirement for PacBio sequencing. In particular, to achieve OD ratios of 1.8–2.0. DNA concentration was determined with the Qubit dsDNA HS Assay Kit (Invitrogen corp., Carlsbad, CA), and high molecular weight content was confirmed by running a Femto Pulse (Agilent, Santa Clara, USA). HMW DNA was sent to Mayland Genomics for HiFi library preparation and sequencing on the PacBio Sequel II.

***Hi-C Sequencing:***

Tissues from the same sample were homogenized using a sterile razor blade on ice. An in situ Hi-C library was prepared as described in Rao et al. (2014) with a few modifications. Briefly, after the Streptavidin Pull-down step, the biotinylated Hi-C products underwent end repair, ligation and enrichment using the NEBNext UltraII DNA Library Preparation kit (New England Biolabs Inc, Ipswich, MA). Furthermore, titration of the number of PCR cycles was performed as described in Belton et al. (2012).

***Reference Genome Annotation***

InterProScan-5.61-93.0 (functional annotation) was run with CDD-3.20, FunFam-4.3.0, PANTHER-17.0, Pfam-35.0, PIRSF-3.10, PRINTS-42.0, ProSitePatterns-2022_05, ProSiteProfiles-2022_05,S MART-9.0, SUPERFAMILY-1.75, and TIGRFAM-15.0 analyses [(Jones et al. 2014)](https://paperpile.com/c/P08Kgf/d6nm). In addition to the BRAKER recommended use of vertebrate orthodb_11 for the proteins, we also used the following 3 reptile annotations: GCF_028583425.1 (*Eublepharis macularius*); GCF_027172205.1 (*Podarcis raffonei*); GCF_027244095.1 (*Hemicordylus capensis*).

**Supplemental Literature Cited**

Belton JM et al. 2012. Hi-C: a comprehensive technique to capture the conformation of genomes. Methods. 58:268–276. doi: 10.1016/j.ymeth.2012.05.001.

Jones P et al. 2014. InterProScan 5: genome-scale protein function classification. Bioinformatics. 30:1236–1240. doi: 10.1093/bioinformatics/btu031.

Rao SSP et al. 2014. A 3D map of the human genome at kilobase resolution reveals principles of chromatin looping. Cell. 159:1665–1680. doi: 10.1016/j.cell.2014.11.021.
